# A Solution for Heritage Monitoring Based on Wireless Low-Cost Sensors and BIM: Application to the Monserrate Palace

**DOI:** 10.3390/s26072015

**Published:** 2026-03-24

**Authors:** Rita Machete, Fábio M. Dias, Diogo M. Caetano, Ana Paula Falcão, Maria da Glória Gomes, Rita Bento

**Affiliations:** 1 CERIS Civil Engineering Research and Innovation for Sustainability, Instituto Superior Técnico, Universidade de Lisboa, 1049-001 Lisboa, Portugal; rita.f.machete@tecnico.ulisboa.pt (R.M.); ana.p.falcao@tecnico.ulisboa.pt (A.P.F.); maria.gloria.gomes@tecnico.ulisboa.pt (M.d.G.G.); 2 Instituto Superior Técnico, Universidade de Lisboa, 1049-001 Lisboa, Portugal; fabio.dias@tecnico.ulisboa.pt; 3 INESC Microsistemas e Nanotecnologias, 1000-029 Lisboa, Portugal; diogo.caetano@inesc-mn.pt

**Keywords:** HBIM, low-cost multivariate sensor, WSN, IoT, monitoring, heritage conservation and management

## Abstract

Conservation and management of built cultural heritage require multidisciplinary approaches and reliable information to support decision-making. In this context, digital transformation strategies that combine Building Information Modeling (BIM) with monitoring technologies offer significant potential to improve heritage management. This paper presents a monitoring solution based on a wireless network of low-cost Internet of Things (IoT) sensors integrated within a Heritage Building Information Model (HBIM), applied to Monserrate Palace in Sintra, Portugal. The proposed approach covers all implementation stages, including HBIM development from as-built data collection, deployment of a wireless monitoring network for acceleration and environmental parameters, and integration of monitoring data into a BIM-based platform. The system aims to create a Digital Shadow of the building as a step towards a Digital Twin framework, enabling centralized visualization and management of structural and environmental information through the HBIM model and dedicated dashboards. Given the lower accuracy of low-cost sensors, in situ calibration with reference equipment was conducted to validate the recorded data. Implementing monitoring systems in heritage contexts presents challenges, such as limited historical documentation and the need for minimally invasive interventions. Despite these constraints, the proposed solution demonstrates the advantages of integrating monitoring data within HBIM, enabling centralized data management and improved understanding of building performance and conservation needs.

## 1. Introduction

Built heritage is a vital component of cultural heritage, playing a central role in industry practices and scholarly debates on tourism; it also strongly influences public awareness and local economic revenue [1]. In this context, preserving and safeguarding built cultural heritage remains a constant priority for institutions, as these structures profoundly shape cultural identity. The practices for conservation, strengthening, and restoration of built heritage require a multidisciplinary approach that encompasses various fields of knowledge, including history, architecture, engineering, and surveying [2,3]. Grasping the background, stages of construction, materials, and shape of the structure is crucial information to acquire before the building’s evaluation and potential restoration efforts [4]. Comprehensive knowledge of each heritage building is essential and should be available to different technical experts; therefore, it is critical to provide the resources and tools needed to enhance the efficiency of management and maintenance [5]. The process of digitalization provides significant advancements across all aspects of life worldwide, and in the construction sector, the adoption of digital dematerialization in process management enables the optimization of fragmented, splintered workflows [6]. The digital transition improves conservation and management practices, requiring a multidisciplinary approach that results in demanding practical applications.

The implementation of a HBIM (Heritage Building Information Model) has immense potential to revolutionize the preservation of cultural heritage when it encompasses data acquisition, 3D modeling, visualization, documentation, monitoring, and simulation. This compounded approach presents a unified data management strategy that enables informed decision-making and predictive maintenance, which, under other conditions, would not be possible. Since the BIM (Building Information Model) does not inherently incorporate monitoring features or automatic data and simulation updates [7], the proposed solution needs to combine strategies from Industry 4.0 to connect an Internet of Things (IoT) system with a Digital Twin (DT).

In the AEC (Architecture, Engineering, and Construction) context, the Digital Twin paradigm encompasses digitized models of physical assets that enable data transmission in at least one direction and real-time monitoring of the asset [8]. Depending on the level of data integration, the term Digital Shadow (DS) appears for models with a unidirectional automated data flow between the physical and the digital; while DT involves a fully automated data flow, where the digital impacts the physical world [9]. The geometric detail of the digital model is a topic of discussion in the scientific community; however, it should align with the purpose of the work or asset being monitored [10], ranging from no geometric representation to conceptual models, to approximations with partially defined elements, to precise 3D geometry, or to as-built representations. For heritage facility management (FM), multiple authors have emphasized the role of BIM as a helpful resource for effectively aiding in the management of built cultural heritage across various tasks, while serving as digital replicas and a centralized database, facilitating not just the geometric representation but also the connection of information with specific components [11]. For an accurate representation, as-built data acquisition—including geometric and material surveys, in conjunction with historical records—is essential to characterize the object of study. Implementation remains challenging due to their intricate and irregular shapes—which affect BIM construction—and heterogeneous levels of information—which limit modeling practices and result in a heterogeneous digital model. In contrast to new construction projects, in the case of heritage, element information may need to be inferred from other surveyed elements or historical counterparts, resulting in components with variable levels of geometric and information confidence within the same model.

As for monitoring, IoT technologies can revolutionize conventional techniques for gathering information in the construction sector, enabling the collection of large volumes of data. With the decrease in the prices of digital sensors and the ability to handle large streams of data, the use of affordable sensors opens new opportunities for facility management [12]. Wireless sensor network (WSN) technology has gained prominence in the management of built environmental assets post-construction due to its potential for minimal-intervention installation (since it does not require cable setup). A WSN is a wireless network of spatially distributed, autonomous devices or sensors that provide real-time monitoring of physical or environmental conditions [13]. Additionally, the benefits of merging the IoT with BIM have been extensively acknowledged in various studies, as this combination can overcome the shortcomings of both [14]. In the cultural heritage scope, museums are nowadays equipped with indoor environmental sensors, typically for collection protection [15,16]. However, major challenges arise with built heritage due to difficulties in installing sensors non-invasively and in ensuring real-time integration of sensor data into a 3D platform. Also, in a heritage building, due to the masonry construction typology with thick walls, the wireless network needs to be correctly dimensioned to account for signal interference from physical obstacles. Likewise, power input for the sensors needs to be carefully considered, including the degree of maintenance required when batteries are employed or limitations on placement when electric current is provided by the building’s grid.

Nonetheless, currently, challenges related to the lack of long-term monitoring strategies remain a concern for implementation. Also, the non-existence of standardized policies and guidelines for HBIM in heritage conservation represents a gap in the literature [17,18]. Both impacted the practical implementation in the Monserrate Palace in Sintra, Portugal. The present paper discusses a low-cost WSN IoT implementation with HBIM, developed within the doctoral research of Machete [19], and explores an integrated multivariable (acceleration and environmental) sensor network within HBIM. This process covers all phases of implementation, from HBIM creation to in situ data acquisition and monitoring network development, including data acquisition, integration, and visualization.

## 2. Literature Review

The National Institute of Building Standards (NIBS) describes BIM as a digital representation of a facility’s physical and functional characteristics, resulting in a shared knowledge resource that serves as a reliable basis for decision-making throughout its life cycle. Despite the various challenges, employing BIM offers several benefits for maintaining and managing built heritage. These benefits include creating a centralized repository for all information related to the building, capable of responding to queries, where data formats and parameter typologies can be used to describe both qualitative and quantitative aspects of the building or its elements. It also allows for the recording of construction interventions, supports analysis, and facilitates maintenance initiatives that require a collaborative, multidisciplinary approach, as highlighted in the literature.

One of the great challenges in implementing HBIM is the lack of information, both graphical and non-graphical. Heritage buildings are often complex and irregular, with limited documentation of materials and their properties compared to new construction projects [18]. To accurately proceed with the digitalization of a heritage site, first, a record of all existing relevant information needs to be collected and cataloged, followed by the characterization of the current state of the heritage building, both geometrically and materially [20]. As such, this task included the collection of all historical records, as well as the procedures and preparations necessary to carry out the fieldwork, such as studying available graphic documentation, conducting site visits, and identifying survey spots to optimize procedures. This is followed by geometrical data acquisition, then the characterization of the constructive elements, concluding with the geometric modeling of all relevant components, to a level of detail and information substantial for the study, in a BIM environment. This requires a survey campaign to enrich and guide modeling practices. These options range from traditional techniques such as classical topographic methods and laser scanning (LS) to image-based 3D reconstruction, such as photogrammetry, which generates a 3D point cloud that facilitates quick data collection on geometry and offers a high degree of precision [5,21]. Also, to determine, with some level of confidence, the approximate characteristics of the structure, in situ experimental tests, both semi-destructive and non-destructive, help bridge the knowledge gap. Depending on in situ limitations and available resources, these tests can include removal of masonry samples, flat-jack tests, ambient vibration tests, ground-penetrating radar (GPR) tests, and thermographic imaging [4,22]. As for modeling practices, the degree of complexity in element representation in HBIM should be determined by its purpose and the analyses to be performed. Therefore, it is essential to determine the geometric details during the modeling process and to identify the pertinent information about the object. Currently, efforts are underway to realize the BIM concept, constrained by available tools and methods, focusing on specific aspects of BIM that only partially fulfill the intended goals. Software comes with its own constraints, and processes frequently fall short; moreover, the advancement of software has not kept pace with the rapid growth of BIM [23].

For safeguarding built cultural heritage, concerns include evaluating its structural integrity against various internal and external influences and monitoring abnormal environmental conditions that may affect the safety and comfort of users or the structure [24]. The monitoring system depends on the type of measuring instrument, sensor, or actuator connected to the IoT system. The implementation of such procedures requires the use of several types of equipment, techniques, and sensors and involves three stages: signal collection, processing, and interpretation [25]. Sensors capture data and collect information from the environment (the conversion of physical events or characteristics into electrical signals), while actuators are responsible for transforming electrical signals into tangible actions (this might be pneumatic, hydraulic, electric, thermal, or magnetic) [26]. In various Internet of Things applications, it is necessary to use one or more sensors to gather data and details about the system. This information is analyzed, and commands might be sent to triggers, which then influence the system. Additionally, the sensors gather data that are sent over the network, along with the actuators that enable device operation [25]. The communication technology used is crucial for the reliable and secure transfer and storage of large amounts of data collected by sensors. It is anticipated that these technologies will become increasingly important in the future as smart buildings and grids become more integrated [27]. Given the wide variety of existing solutions, defining monitoring systems can be complex. Inadequate instrumentation may result in undesirable outcomes, including misreading of data and incorrect conclusions, stemming from potential information loss or inadequate signal processing techniques. Therefore, understanding the key attributes of the sensors employed is a valuable advantage, as this understanding helps make the most appropriate sensor choices and implement them [28]. The type of equipment determines the type of solution applied. In this context, open-source tools and custom sensor systems are particularly favorable, as they can be fully tailored to specific application needs without dependence on third-party platforms, paradigm shifts, or product discontinuation. A self-sufficient IoT system for continuous monitoring—with minimal labor involvement through non-destructive testing principles—as well as kinematic and environmental sensors with dynamic responses, most relevant for structural health monitoring (SHM) and indoor environmental monitoring (IEM).

Structural health monitoring (SHM) has expanded markedly in recent decades, particularly for assessing seismic performance through the integration of monitoring, inspection, and analysis. Experimental dynamic characterization supports accurate condition assessment, numerical model calibration, and retrofit design. Although IoT-based continuous SHM is still emerging, long-term monitoring enhances real-time decision-making, improves reliability in performance prediction, and supports timely maintenance [29,30]. Indoor environmental monitoring (IEM) also has a sizable impact, especially on energy consumption efficiency and comfort. The demand for effective, innovative, energy-efficient solutions has grown amid global efforts to reduce carbon emissions and fossil fuel dependence [31]. In IEM, monitored variables support facility management and building optimization, covering occupancy, water, and energy (e.g., HVAV and artificial lighting) consumption, as well as indoor comfort conditions. Key parameters include temperature, relative humidity, indoor air quality, light, and noise. IoT sensor data, organized by location or asset type and linked to business rules, enables algorithms to detect and respond to anomalies [32]. Continuous monitoring using budget sensors, often integrating WSN and BIM, has been reported for historic buildings [33], museums [34], and facility management [35]. Post-construction environmental data use for energy savings is also widely studied [36].

The integration of HBIM with IoT requires as-built surveys, simplified modeling, and data integration. This integration is crucial, as a lack of access to information on the buildings’ current state makes it difficult to implement an active management strategy and plan appropriate interventions. In the literature, applications can be found at various implementation scales. Desogus et al. [37] demonstrated a proof-of-concept process for a common data platform to visualize indoor building conditions in two rooms using a Dynamo script to connect to the IoT platform in the Mandolesi Pavilion of the University of Cagliari. Martinelli et al. [38] explored an open-source data platform integrating an HBIM–IoT monitoring sensor web server that was focused on environmental monitoring (not carried out directly by the authors) of the “Camera Oscura” at the Bourbon Royal Site of Carditello. In contrast, Dionizio et al. [39] implemented a full system for one sensor, from monitoring to data management via OneDrive, installed in the MCK (Modern Ensemble of Pampulha).

## 3. Materials and Methods

**Figure 1:**
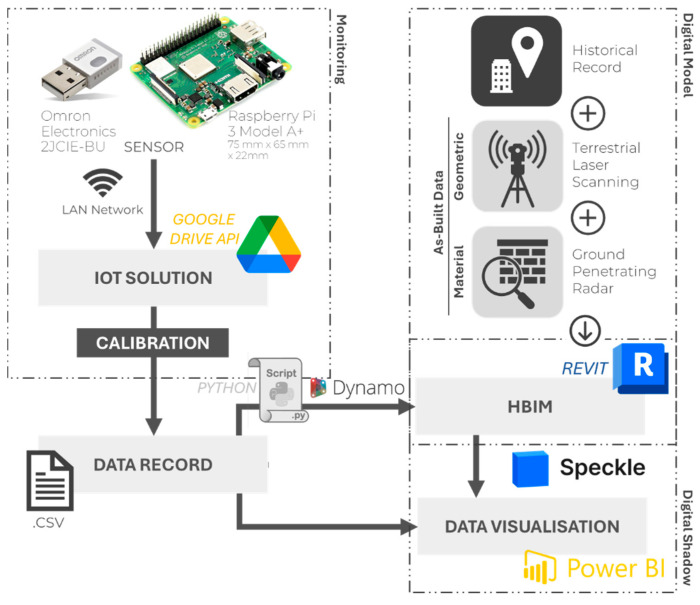
Workflow-integrated multivariable IoT sensor network and HBIM.

Current research identifies a gap in integrating HBIM and IoT to monitor environmental conditions and support preventive conservation of collections in historical buildings. This demonstrates a clear need to present and discuss additional implementations by using diverse methods and tools to advance research and applications in the field, particularly for heritage buildings whose inherent complexity and heterogeneity demand an individual basis approach. The current research aims to address this gap by presenting a workflow (Figure 1) for an integrated multivariable (acceleration and environmental) IoT sensor network in HBIM while considering all phases of the process, from data acquisition to integration. This process culminates in the integration of sensor records into a common data platform for the visualization of building conditions, both in a model and through dashboards. The integration of real-time sensor data with the 3D digital model (HBIM) results in a Digital Shadow with a strong graphical component that enables an expedited understanding of the site, while at the same time identifying abnormal conditions and supporting pattern recognition over time.

As a base, a digital model representing the heritage building and serving as a centralized database needs to be constructed, incorporating historical records, geometric surveys, and material characterization. For monitoring, a low-cost multivariable sensor module was selected due to several advantages. These include its lower price point, which allows the deployment of a dense sensor network for an extensive characterization of the case study; its versatility across a wide range of applications, enabling the simultaneous registration of multiple physical and environmental quantities; the availability of commercially supported connectivity options that facilitate an IoT-based wireless solution, thereby minimizing installation impact on the heritage building and enabling remote data handling; and its small form factor, which allows the integration of multiple sensing elements within a compact sensor unit. Notwithstanding these advantages, and due to inherent equipment limitations, parallel monitoring using well-established measurement systems remains essential for data validation and calibration.

### 3.1. Data Collection and BIM Modeling

**Figure 2:**
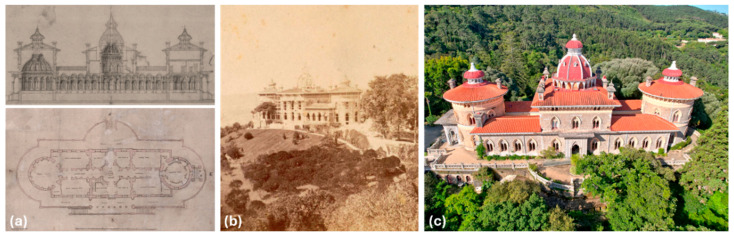
Historical record: (**a**) 1858/1864 drawings by James Knowles ©CMS, (**b**) 1893 photo by Francis Cook ©PSML, and (**c**) northeast facade by Andre Dias ©PSML.

Monserrate Palace (Figure 2) is part of the cultural landscape of Sintra and has been a UNESCO World Heritage site since 1995. The current iteration of the building dates to 1863, commissioned by Francis Cook to the architects James Thomas Knowles, senior and junior, for the reconstruction of the main house, which had previously fallen into ruin (De Visme neo-Gothic castle). The site shows discontinuous use and management, leading to several significant interventions over the centuries and periods of ruin from neglect and natural disasters, ranging from the destruction of the 1755 earthquake to property abandonment in the 1790s, and again in the 1900s, to a major storm in 1983. The neglect of the site was addressed through rehabilitation works from 2000 to 2010, and the management of the property was handed over by the Portuguese state to “Parques de Sintra-Monte da Lua S.A.” (PSML) in 2007 to allow for continuous care [40]. Currently, the palace functions as a museum, with sporadic use as a conference or entertainment space, and is characterized by its residential, eclectic, revivalist architecture, known for its stucco decorative elements. The building is a self-supporting structure that rests on a raised platform, with a longitudinal, symmetrical plan along the axis of the main corridor, a rectangular central body, and two circular-plan towers at the ends. The building is 60 m long by 20 m wide, with 5 floors. Most of the floors comprise wood flooring supported by wooden beam structures, with a few exceptions of tile flooring over brick vaults supported by steel beams. The exterior (structural) walls of the building are rubble stone masonry with air lime mortar, while the interior walls are “tabique” partition walls. However, due to the lack of detailed records, the composition of all existing elements is not immediately evident; for the extensive characterization of the monument, it was necessary to execute non-destructive tests to ascertain the constitution.

**Figure 3:**
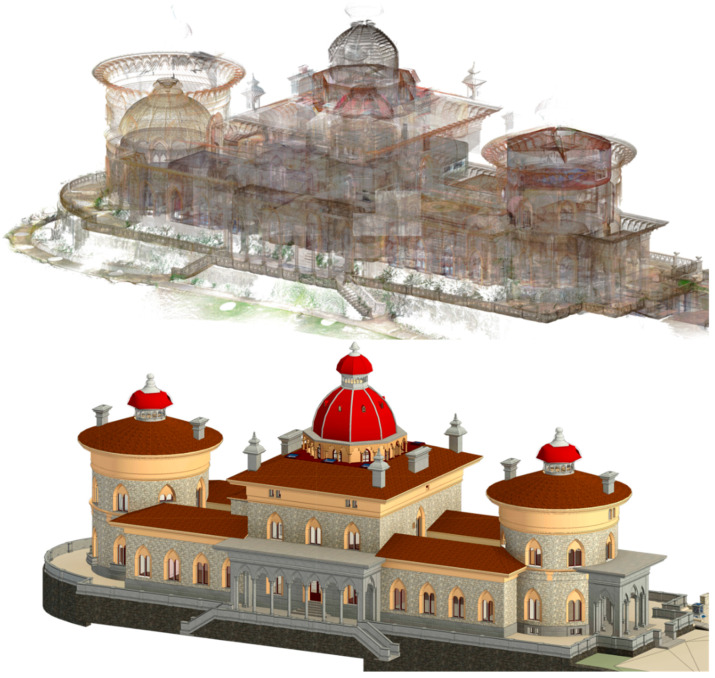
As-built geometric data collection, (**top**) point cloud from TLS, and (**bottom**) HBIM perspective southwest view.

To this end, ground-penetrating radar (GPR) surveys were conducted by the Morph Geociências, Lda team, coordinated by the Instituto Superior Técnico team. Therefore, in areas where the construction technique was obscure, the technique was applied by emitting and receiving electromagnetic waves from the object, allowing the deciphering of cavities and material alterations, which permits a better understanding of the constitution of the structural elements. This inspection method is non-intrusive and therefore subject to interpretation; nonetheless, the construction apparatus can be examined in the radargrams, where the pattern correlates with a construction methodology. A total of 22 3D polygons and 9 discrete profiles were inspected, and the morph team interpreted the information collected. Due to the extensive work that the palace underwent throughout its lifetime, a comprehensive geometric survey is crucial to accurately reproduce current conditions. To capture the current state of the building, a geometric survey was conducted using a Faro Focus S70 (FARO Technologies, Lake Mary, FL, USA), a phase-based terrestrial laser scanner (TLS) that emits a continuous, modulated near-infrared laser signal (1550 nm) and determines the distance to objects by measuring the phase shift between the emitted and reflected signals. A total of 336 scans were conducted, with the profile type selected in accordance with the scan position—distinguishing between interior and exterior spaces—and the area captured. The majority of scans occurred indoors, with relevant features within a 10-meter range. For the roof structure, due to the building’s symmetric characteristics, scan positions on the top balcony provided sufficient information for modeling, which was validated by drone imagery. To process the scans, the Autodesk ReCap 2023 (Version 23.1.1.354) software [41] was used. This is a 3D program for complex laser scanning and photogrammetry projects, which processes the file formats acquired from various equipment to create an ordered point cloud. Following this, it allows for the export of files in the proprietary Autodesk format, point cloud project file (RCP), which integrates seamlessly into other software such as Autodesk Revit 2024 (Version 24.2.0.63) [42], which is necessary for the creation of the HBIM of the palace. To maximize software performance and to guarantee positional accuracy, the scans were organized into 15 clusters (3 for the exterior areas, 4 facades, and 8 interior zones). The final point cloud, after cleaning and being unified (unstructured), has ≈753,740,000 points (exported with 5mm spacing) of ≈17 GB, which uses fewer computational resources but still has the relevant features (Figure 3, top). With historical, material, and geometric as-built information collected, the HBIM can be constructed (Figure 3, bottom). When creating a digital model, the geometric representation of the building must be compatible with its intended use at an appropriate level of detail. For heritage buildings in a conservation context, information on materials and elements must be registered with a high level of detail, either as attributes or in geometry. In the case of BIM-based management, the level of detail can be simplified, while the attributes should support operations throughout the building’s life cycle. The present model has more than one intended use: acting as a centralized database of data and information, functioning as a basis for a Digital Shadow, and encompassing future monitoring aspects (of management).

### 3.2. WSN Data Acquisition, Storage, and Management

Continuous monitoring was implemented to assist in the management and preservation of the palace. The equipment employed included a multivariable sensor unit—the OMRON Electronics 2JCIE-BU—which, according to the manuals [43,44], is an environment sensor USB Type (length 29.1 mm × width 14.9 mm × height 7 mm; weight 2.9 g) that establishes connectivity via Bluetooth low energy (Bluetooth v5.0) and USB2.0, offering a communication range of approximately 10 m. The operating temperature ranges from −10 to 60 °C, and the relative humidity is 30 to 85% RH. Measurement of kinematic quantities took place via 3-axis acceleration, alongside environmental quantities, including temperature, relative humidity, ambient light, barometric pressure, sound noise, eTVOC (equivalent Total Volatile Organic Compound), and eCO_2_ (equivalent CO_2_). The detailed range of the sensor extractable parameters is described in Table 1. On the other hand, the OMRON USB sensor requires connectivity, programming, data storage, and a power supply, which can be provided through a Raspberry Pi. The version used was the Raspberry Pi 3 Model A+, with a 64-bit quad-core processor running at 1.4 GHz, dual-band 2.4 GHz and 5 GHz wireless LAN, and Bluetooth 4.2/BLE. The system used a 32 GB microSD card for operating the system installation and data storage. Power was supplied by a 5 V, 2.5 A DC source via a micro-USB connector.

One objective was to create a highly scalable, distributed network, with each Raspberry Pi node equipped with a dedicated sensor. This process was conducted through testing, data management evaluation, and calibration. The creation of this system involved several iterations, one of which was presented at the 2024 IEEE SENSORS conference [45]. To configure a Raspberry Pi node, the official operating system was first flashed onto the microSD card (using Raspberry Pi Imager v1.8.5), which serves as the primary storage medium. Subsequently, essential packages and utilities were installed, the LAN network was configured, and custom firmware was deployed. In this step, it also configures the node identifier and the palace’s Wi-Fi network. Because the Raspberry Pi must maintain a physical connection to power the sensor, the USB/Serial interface was selected for data communication instead of Bluetooth. The well-documented information on the communication protocol and available commands, provided by the OMRON Developers Hub and the OMRON Environment Sensor User’s Manual, played a fundamental role in the development of this application.

The custom firmware implemented on each Raspberry Pi node manages data acquisition, event detection, local storage, and cloud synchronization. It automates sensor configuration, timestamp synchronization, and parameter logging. For example, for time synchronization, the Raspberry Pi clock is updated over Wi-Fi; if the connection is lost, the node will not be registered. This guarantees that the data are comparable across the time parameter between sensors. Alerts are triggered when specific parameters exceed or fall below the established bounds. The sensor is equipped with two operation modes, the normal mode and the acceleration logger mode, which can be selected to match the application. Normal mode can save sensing data to the built-in flash memory at an arbitrary interval and automatically save raw acceleration data to the flash memory when a trigger occurs, i.e., during an earthquake or vibration. Acceleration logger mode is designed to measure acceleration and save raw acceleration data at specified operating frequencies and periods. It does not calculate the SI value, PGA, etc. As such, under regular conditions, the sensor records entries every second. Using this information, for the acceleration values, a threshold function was applied. Several types of thresholds are described in the manual, ranging from simple, change, average, and peak, among others. In the present case, when the values exceed the predefined trigger threshold, the ‘logger mode’ is activated and an alert is logged. Due to internal architectural constraints of the sensor, switching between operating modes introduces unavoidable recording gaps. The record will stop, and the records will initiate, now with entries every 0.005 s. A break in data registry will occur as the modes are changed, ≈3 min, the logger mode will register for ≈27 min, followed by a second data break of ≈25 min, and then the records will start again in the base file. This technical limitation may affect the registration of replicas; however, the dynamic behavior post-event would still be recorded. Currently, no solution is implemented; however, pairing sensors at strategic locations to enable continuous recording in logger mode was discussed. This solution would then require the acquisition of more sensors, as well as an impact on data storage capabilities.

For storing and accessing data, a copy of the raw data is backed up on the SD card, allowing for record retention in the event of lost connectivity. As for the IoT solution, Google Drive was used, providing a simple, fast, and secure way to prototype and share data across nodes. This approach allowed rapid development and testing without requiring dedicated server infrastructure. It is important to note that Google’s free service has a storage limit of 15 GB. Since part of the acquisition rate is event-driven, it is not possible to accurately determine the amount of data per period of time. However, during the operations presented in this work, an estimated 100 MB of data is produced by one sensor in normal mode every 13 days. However, as the volume of data from each node grows, this solution has begun to face limitations, including storage quotas, limited structured querying, and scalability constraints. Future implementations should therefore consider migrating to a dedicated database backend. Relational databases (e.g., PostgreSQL) or, more appropriately, time-series databases (e.g., InfluxDB or TimescaleDB), would provide efficient management of high-frequency sensor data, support real-time analytics, and enable scalable deployment across larger sensor networks. Also, for efficient data management, for environmental data (such as temperature), a 10 min average should be sufficient to characterize the spaces (see [46]); as for acceleration, the logger mode records better describe the building response; however, the trigger information remains in the base record.

During operation, Google Drive files are updated every 20 min to avoid overloading the Wi-Fi network. To further reduce the number of simultaneous connections, the update time is randomized across sensors. To support data handling, in previous iterations, new records were added to the same CSV file, making it difficult to open due to RAM (Random Access Memory) limitations. In this case, the data are divided into new files once 20 MB is reached. This also allows software such as Microsoft Excel spreadsheet to open the complete file (row limit of 1,048,576 in Microsoft Excel spreadsheet).

### 3.3. Sensor Calibration

To validate the record data from the sensor unit (the OMRON USB sensor and Raspberry Pi node) against an established sensor, testing was conducted in situ (in the palace) and under more stable conditions. Therefore, for environmental monitoring tests, three quantities were tested with unique dynamic monitoring. The quantities tested were temperature (°C), relative humidity (% RH), and illuminance (lux), conducted between an established sensor, the ONSET HOBO U12-012 (ONSET, Bourne, MA, USA), and the OMRON 2JCIE-BU (OMRON, Osaka, Japan). These devices have similar characteristics and prices, as described in Table 2, but in the case of HOBO, data storage is only local, and it is a battery-operated device (with no power outlet connection), which may be a factor for extended continuous monitoring campaigns.

**Figure 4:**
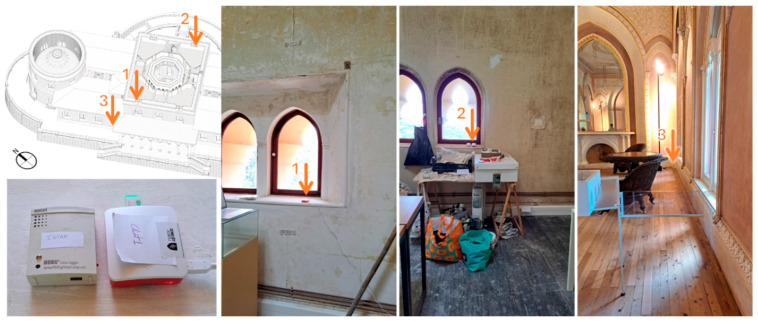
Equipment position in the palace, in situ survey from 8 April to 16 July 2024, OMRON and HOBO; positions 1 and 2 are on windowsills on the second floor (southwest and northeast workroom), and position 3 is in a wood floor corner on the ground floor (dining room).

A test was conducted in the palace at three positions from 8 April to 16 July 2024, with the HOBO programmed to register at a 10 min logging interval to compare with the raw data records for the OMRON (at 1 s intervals), for which the average value was calculated at a 10 min interval for better comparative calculations. The locations of the sensors are shown in Figure 4, with two sets on the second-floor windowsill of the southwest and northeast workrooms, and one set on the ground-floor dining room, placed in the corner of the room near the exterior wall.

**Figure 5:**
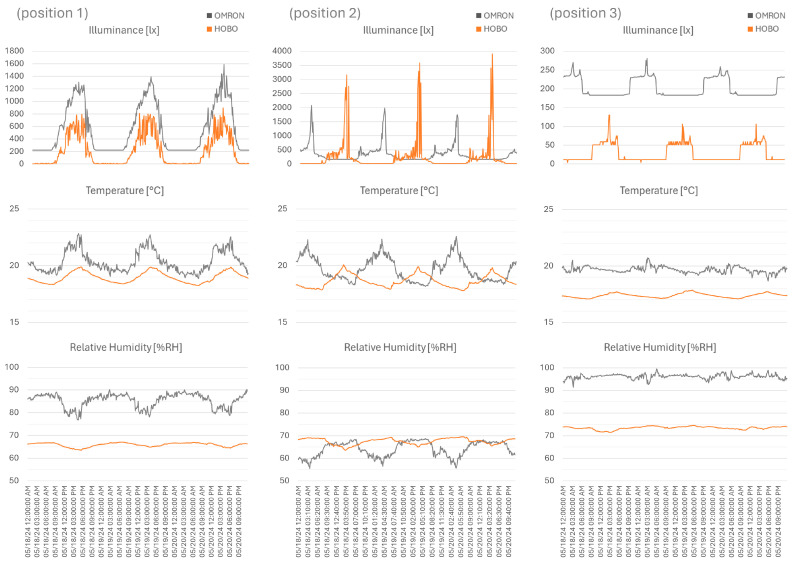
Illuminance (lx), temperature (°C), and relative humidity (% RH), from 18 to 20 May 2024, for HOBO and OMRON (average 10 min) for the three positions.

To facilitate the interpretation of the graphic data, data were selected for the period from 18 to 20 May 2024 for illuminance, temperature, and relative humidity; see Figure 5. Evaluating the data excerpt, an internal clock misalignment is apparent for positions 2 and 3. To facilitate coherence among the three sensor clocks, in the latter implementation, the record time information is updated from the Wi-Fi connection.

Examining the illuminance, both sensor data samples show the same trend across the three positions, yet a positive shift is observed. The data range also varies significantly, with a range error of ≈50% for positions 1 and 2 (windowsill; therefore, higher levels of sunlight) and ≈20% (room corner; lower light levels). This variation between data may occur due to the sensitivity (accuracy and precision) of each sensor. Also of note is the OMRON sensor’s ±100 lx accuracy, which influences the data.

For temperature, as with illuminance, the same trend is observed, although a positive offset is evident in the OMRON data. The accuracy difference needs to be noted, as it has a significant impact on records; therefore, the HOBO has ± 0.35 °C and the OMRON has ±2 °C accuracy. The bias between units is 1.4 °C, 1 °C, and 2.3 °C, representing the difference between averages and the value in proximity to the sensor accuracy threshold. This also affects the range in recorded data, with OMRON sensors presenting ≈4 °C for positions 1 and 2 (windowsill is more exposed) and ≈2 °C (room corner is less exposed), in relation to ≈2 °C and ≈0.75 °C for the same positions in the HOBO sample.

As for the relative humidity, the offset in the data is inconsistent across the three sensors: a positive shift for positions 1 and 3 and a negative shift for position 2. The bias between units is 19.5% RH, −3.5% RH, and 27.7% RH, which is the difference between averages. Acknowledging the difference in accuracy is crucial for evaluating the samples; thus, the HOBO has ±2.5% RH and the OMRON has ±5% RH accuracy. The sample range in the OMRON sensors is ≈13% RH for positions 1 and 2 (windowsill is more exposed) and ≈8% RH (room corner is less exposed). As for the HOBO, positions 1 and 3 have a range of ≈3% RH, while position 2 shows a larger range of ≈6% RH. The amplitude of the data is once again explained by the sensor’s accuracy; however, this does not excuse the recorded bias.

**Figure 6:**
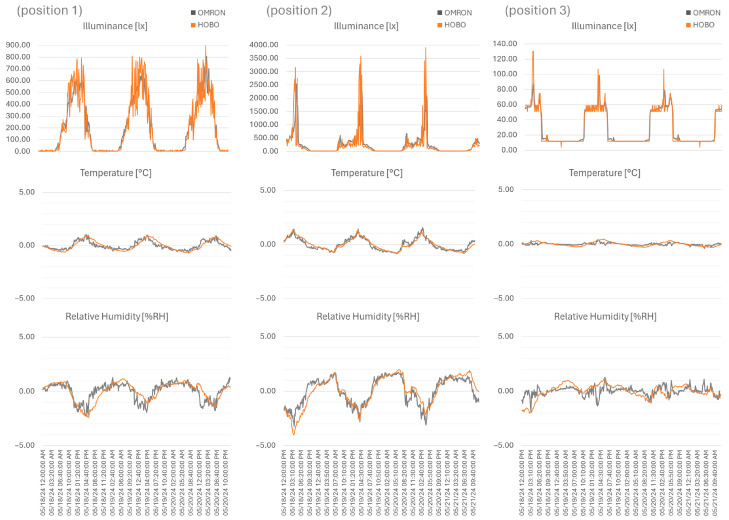
Illuminance (lx), temperature (°C), and relative humidity (% RH), from 18 to 20 May 2024, for HOBO and OMRON (average 10 min) for the three positions, synchronized by features and linear calibration (offset and gain correction).

For data analysis, the clock misalignment was corrected, the data were synchronized using features, equivalent peak values were identified, and the time alignment for the x-axis was determined. Then, a linear calibration (offset and gain correction) was implemented for the y-axis (Figure 6). For illuminance, the OMRON records were shifted by the minimal value of the HOBO, representing night-time conditions; while, for temperature and relative humidity, both sets were shifted by the average to zero. A gain compensation value was then reached from iterative testing, determined when the RMSE (Root Mean Square Error) value was at its lowest, measuring the average magnitude of the error between predicted and actual values. Following the position order, the gain value for illuminance was 1.7, 0.76, and 1.13, altering the RMSE from approximately 395 lx to 84 lx, 427 lx to 410 lx, and 173 lx to 7 lx, respectively. For the temperature variable, the gain was 2.4, 1.85, and 2.6, altering the RMSE from approximately 1.5 °C to 0.25 °C, 1.2 °C to 0.2 °C, and 2.3 °C to 0.2 °C, respectively. For relative humidity, gain was 3.75, 2.7, and 2.6, altering the RMSE from approximately 19% RH to 0.6% RH, 4% RH to 0.6% RH, and 22% RH to 0.6% RH, respectively. To evaluate the impact of the selected offset and gain, the RMSE values were compared for data 1 and 4 weeks after the initial evaluation. Some variation was identified, but the correction values continue to prove adequate; however, a future recalibration campaign may be advantageous. The illuminance readings over the specified time intervals were as follows: sensor 1 demonstrated a RMSE variation from 84 lx to 72 lx to 124 lx, sensor 2 displayed variations from 410 lx to 367 lx to 262 lx, and sensor 3 showed values from 7 lx to 7 lx to 5 lx. However, the temperature varied: for sensor 1, it ranged from 0.25 to 0.6 to 0.7 °C, for sensor 2, it ranged from 0.2 to 0.7 to 1.5 °C, and for sensor 3, it ranged from 0.2 to 0.3 to 0.7 °C. Furthermore, relative humidity varied: for sensor 1, it ranged from 0.6% RH to 0.9% RH to 1.5% RH; for sensor 2, it ranged from 0.6% RH to 0.8% RH to 1.5% RH; for sensor 3, it ranged from 0.6% RH to 1.6% RH to 1.5% RH. The squaring process in the RMSE results in assigning a disproportionately higher weight to larger errors, making it sensitive to outliers, as is embodied in the illuminance dataset of position 2. The OMRON sensor displays higher peaks, resulting in a difference between measurements; this occurrence highlights the difference in sensitivity between units, also displayed in the significant gain correction value.

For the OMRON sensor, it was observed that the accuracy for each quantity has the largest impact on data confidence. The offset in data may be the result of lower precision in the OMRON sensor in comparison to the HOBO. The sensor resolution, as well as the minimal value of range, may lose relevance if the accuracy does not follow the same scale.

**Figure 7:**
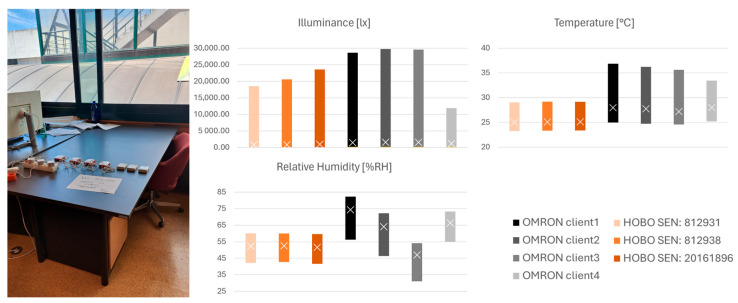
Equipment office test: position in room, from left to right, OMRON 3, 2, 1, and 4, and HOBO 812938, 812931, and 20161896. Plot Max–Min–Avg from 10 min data average, from 12 to 13 September 2024, for OMRON and HOBO (x average).

Due to some inconsistency, a third test was conducted, with the same characteristics as the second test; therefore, all equipment was tested under the same conditions. The OMRON sensors (1 to 4) and HOBO sensors (812938, 812931, and 20161896) were placed on a table facing a westbound window on the third floor in Instituto Superior Técnico at Rovisco Pais Avenue, Lisbon (Figure 7). A sample of data was selected for the 12 and 13 of September 2024. For error calculations, the HOBO Sensor 20161896 was used, as it is the most recently acquired. As it occurred during the palace test, all data followed the same trend, again presenting the same obstacles regarding sensor accuracy. The HOBO sensors recorded reasonably the same values, apart from illuminance, for which some of the recorded variance for the HOBO and OMRON sensors may be accounted for by the effect of the shadow from the window frame. Once more, a positive shift for the temperature values was recorded for all OMRON sensors, with an average difference of ≈2.5 °C and a range twice the size (range for HOBO ≈ 5.8 °C; OMRON ≈ 11 °C) as it occurred in the palace. For illuminance, the minimal value in the OMRON sensor was once again higher than the accuracy value (c1 195 lx, c2 219 lx, c3 187 lx, and c4 187 lx) in comparison to the HOBO, which reached a minimal value of 4 lx. As for the maximum value error, it was ≈25%, excluding sensor 4. Irrespective, the OMRON client 4 appears to register fewer extreme values and lower peaks than both types of equipment. As for the temperature and relative humidity ranges, compared to the other OMRON, the data record was closer to the HOBO values, with a range error of 42% and 2%, respectively. The difference between this sensor and its counterparts reduces with a larger dataset.

For the present sensors and the current work, the inconsistencies were considered within an acceptable range, except for the relative humidity (% RH) parameter, for which the values exhibited unpredictable shifts between sensors. To correct this deviation after logging, when employed in situ, to determine the standard value for correction, the pre-established sensor was placed alongside the node. In the present case, the HOBO equipment was deployed, registering at 1 s intervals to mirror the OMRON raw data.

For the ambient vibration tests, a previous iteration of the work, reported in [47], evaluated the reliability of a low-cost MEMS sensor by comparing it with a force-balance accelerometer system, the ETNA2 (EpiSensor ES-T triaxial sensor with a digital recording unit) manufactured by Kinemetrics Inc. The results showed that similar vibration frequencies were obtained when the data were analyzed using the ARTeMIS Modal software (Version 7.2.1.5) [48]. However, due to its higher sensitivity, the ETNA2 sensor produced clearer spectral peaks, facilitating the identification of vibration frequencies (or periods) and the corresponding modal shapes. Continuous monitoring also enabled event detection and trend analysis, including the identification of changes in vibration behavior associated with restoration works. Based on these findings, the in situ tests carried out for the OMRON sensor configuration focused on establishing the baseline signal amplitude under typical operating conditions, as well as the corresponding noise level. These measurements were used to determine an appropriate threshold for activating the logger mode, enabling reliable event detection and subsequent trend analysis.

Noise limits detectivity; in other words, it limits the lower bound of the dynamic range. MEMS accelerometers are inherently more susceptible to internal noise [49]. With premium equipment, such as the ETNA2 or equivalent force-based accelerometer, noise is minimal, allowing the creation of pre-established acceleration thresholds that are typically set at ±0.001 g to automatically trigger recording [50,51]. Thus, sections of data acquired in an ordinary condition in ‘normal mode’, that is, measuring at a 1 s period (which is the usual sensor state that will determine when the threshold is activated), were analyzed to determine the signal amplitude. To determine the influence of sensor position on the impact of vibration results in the palace, two sensors were used in this test: one sensor was positioned on the floor in the drawing room on the ground floor, aligned with the stairway supporting wall, and another was positioned on the windowsill (in the masonry wall) on the second-floor workroom.

**Figure 8:**
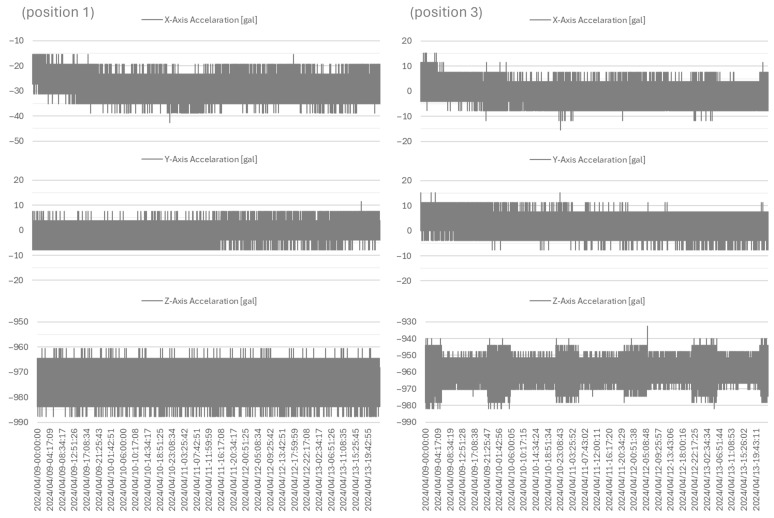
OMRON accelerometer data from 9 to 13 April 2024 (position 1) for the windowsill in the masonry wall, and (position 3) wood floor system.

In the case of the XYZ accelerometers, their outputs skew off from zero if their position has a slight tilt, with respect to the vector of Earth’s gravity, and therefore feel some pull [52]. As such, if the sensor is perfectly aligned, the X- and Y-axis signals will present around 0 value, and the Z-axis signal will be 1 g, or ≈980 gal, representing gravity. Figure 8, representing a section of data, allows for evaluating the amplitude of the signal in ordinary conditions; it can also be observed that a baseline shift naturally occurs in the raw data (a detrending operation may be advantageous for some data analysis). From Figure 8, it is clear that, although not stated in the datasheet, the effective resolution of the OMRON sensor for acceleration is limited by the quantization step that was experimentally estimated to be 5 gal. Additionally, the noise level appears to be in the same range, as it produces very few random-like oscillations in the measured signal through day-long acquisition. Therefore, in the masonry wall for the X-axis, the greater amplitude is ≈19 gal; in the Y-axis, the greater amplitude is ≈15 gal; and in the Z-axis, the greater amplitude is ≈27 gal. As for the sensor on the wood floor system, for the X-axis, the larger amplitude is ≈19 gal, with segments of ≈15.3 gal; in the Y-axis, the greater amplitude is ≈15 gal; and in the Z-axis, the amplitude presents two ranges of ≈23 gal and ≈34 gal. As such, to minimize false triggers while still accounting for any incident outside regular conditions, the threshold was set at 30 gal, with the trigger activated by the difference relative to the previous record. This value is specific to this case study and was selected to minimize the trigger of logger mode for unrelated conditions to an event detection (such as walking) since the flooring has high flexibility; therefore, in new positions or another case study, a different value may be adequate.

During the following implementation test, from 23 July to 16 December 2024, it became clear that the established threshold in the Z-axis is regularly triggered by the flexibility of wood flooring. This causes undue strain on data storage management and the data registry since these triggers do not represent risk conditions; therefore, the gap in data records and the extra storage space are not justified. As such, the trigger for the vertical axis was removed, since an earthquake event will affect the XY-axis regardless of the Z-axis.

From the calibration, the limitations of an affordable sensor were detected, such as its lower precision and accuracy compared to high-end equipment for recording the same quantities. This resulted in some deviations in the recorded data; however, they are perfectly capable of capturing trends and variations in the quantities observed.

### 3.4. Continuous Dynamic Monitoring with a Sensor Network

For the implementation of the WSN, the first step was determining sensor placement in the palace. These positions need to take the following into account: (i) power supply, as this solution does not have an internal battery; (ii) fastening mode, which due to heritage concerns, needs to be of minimal impact and reversible; (iii) dispersion through the palace in spaces with possible different characteristics, which is relevant for vibration information; and (iv) orientation to simplify vibration data interpretation. The position of the sensor, specifically the tilt, affects acceleration data (which is why higher-value equipment has a level incorporated).

**Figure 9:**
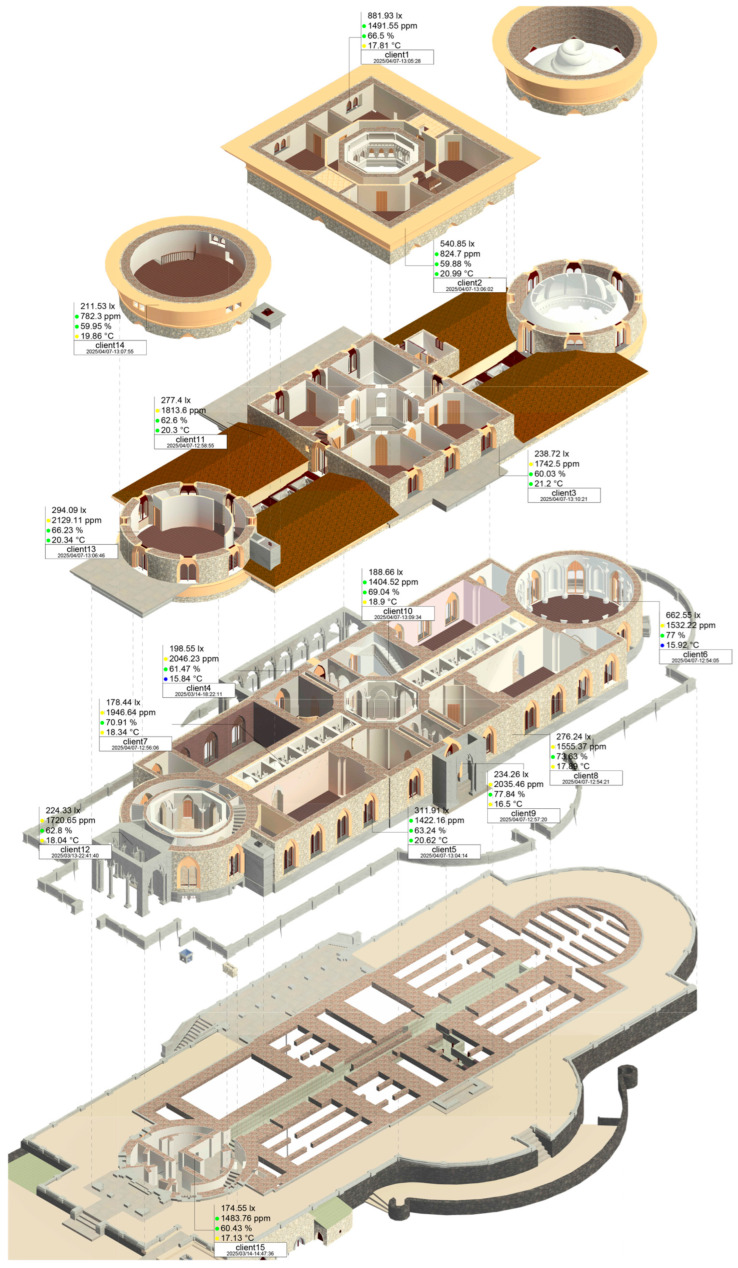
HBIM exploded axonometric diagram with OMRON nodes positions and tags.

In this study, 15 OMRON sensors were placed in the palace (Figure 9), 8 on the ground floor, 3 on the first floor, 3 on the second floor, and 1 in the foundation. The sensor locations were selected to monitor spaces with different solar orientations while capturing the main dynamic characteristics of the palace’s structural bodies. When relevant, sensors were also positioned relative to structural elements to record structural vibrations. For example, sensors 3 to 12 were installed in the palace’s exhibition areas to assess variations related to occupation and orientation; where appropriate, they were placed adjacent to structural walls to evaluate vibration response (sensors 3, 5, 7, 8, 10, and 11). Sensors 1 and 2 were installed on windowsills on the upper floors to monitor structural vibration, sensors 13 and 14 were positioned in the south tower, and sensor 15 was placed at the foundation level to enable a comparison between livable and non-livable spaces. The placement of sensors was either at the room’s corner to reduce the impact of the wood floor system vibration, which also reduced accidental interference from users of the space (be it tourists or cleaners), or at windowsills, to better record the structure vibration on masonry walls. As a fastener, double-faced tape was used, allowing the sensor to remain in place during everyday activities but allowing easy removal during maintenance and the testing phases. For a more permanent solution, a mechanical fastener, like a screw, could be affixed from the case cover to the flooring.

The two major obstacles to sensor network implementation in the area were Wi-Fi connectivity and power supply stability. Due to the wall thickness and density, the original router distribution in the palace was insufficient to reach the whole network. Therefore, the first step was to supplement the router network to guarantee connectivity throughout the palace; without this, WSN would not work. After implementation, the second obstacle occurred due to the power supply’s advanced age, which manifested as power fluctuations that caused sensors to enter the standby mode. When the sensor fully disconnects from power and is reconnected, the equipment will reinitiate; however, the power fluctuation leaves the sensor in a midway point, which requires the manager to go to the site and reinitiate the node. For a future update of the WSN, the inclusion of a battery pack would be advantageous to resolve this problem and minimize data gaps in the records, either due to loss of power from human intervention or in a hazardous situation.

## 4. Results

With the digital model created, WSN employed, and raw data stored in the Google Drive folder, the next steps involve data visualization and the insertion of relevant data into the HBIM model. Two avenues were explored to achieve this: the first being the creation of Python (Version 3.11) [53] and Dynamo (Version 2.19.3) [54] scripts to extract data and insert it into the Autodesk Revit (Version 24.2.0.63) [42] environment; and the second being the facilitation of Microsoft Power BI (version 2.144) [55] dashboards for data visualization, which were created coupled with the Speckle (Version 2.19) [56] platform viewer, allowing a model representation with sensor location visualization.

In the first path, a Python script extracts an average of the last 10 min for each variable, and then a Dynamo script inserts the data into the corresponding element and parameter in the HBIM in Revit. To start, the last file for each node needs to be selected (excluding the ‘acc’ files), and the last 600 lines of the raw data need to be extracted to a new CSV file. Then, the header is inserted, and empty lines are removed. In this file, a script calculates the average for each column, creating a new file with a line per variable and its average. Since the time column does not return a value, the last entry from the data is extracted to determine the time interval for the averages. These scripts were created for each node. Each time the script is run, the data in the CSV files are updated. With the calculation concluded, the data need to be inserted into the Revit model and connected to an element representing the node. The physical dimensions were reproduced, and an XY-axis representation was added to facilitate reading. To allow further representation of the data, for visualization filters and graphic representation in tags, shared parameters were created. With the model accurately placed and oriented in the project, the Dynamo script can be implemented to update the parameters. The element ID of each sensor representation is used to allow the data to be connected to the relevant element and associate the parameter with the last entry for the time and the average data for the quantities. For the relative humidity quantity, the value needs to be corrected, adding the shift value registered from the comparison with HOBO equipment results; therefore, an extra node was created only for this parameter. Due to potential discrepancies between sensor readings and script execution times, the comment parameter was employed to establish a temporal correlation with the model. This node also unlocks the possibility of a periodic script running if the Dynamo script is opened. For this to function properly, the Python script would also need to be automatically run; to this end, a scheduling tool must be used. Regardless, for the HBIM to update, the software needs to be opened.

With the data in the element, the next step is visualization. Since shared parameters were used, these data can be consulted in the properties tab of the respective generic model, in the corresponding schedule, and in a custom-made tag. For indoor comfort assessment, environmental threshold values for temperature, relative humidity, and air quality were determined in accordance with the literature. With the values inserted into the model, it is now possible to create color filters that quickly interpret the measurements observed by the sensors. To represent these ranges in the BIM software, intermediate ‘yes or no’ binary parameters were created that change according to an ‘if’ condition when the value falls within the relevant range.

To understand the quantities recorded, it is important to understand their impact on the management and/or comfort of the user and the building. Measures such as temperature and humidity are relevant, both because of their impact on energy consumption (through their control by HVAC equipment) and for indoor comfort control. These values have added relevance in a museum space, where the risk-free amplitudes must consider not only the user but also the collection on display; these may vary with the season or outdoor temperature. Similarly, due to the risk of acute toxicity, guidelines for indoor CO_2_ concentrations also exist and are relevant to an inhabitable space. To apply filters, value thresholds need to be implemented in line with World Health Organization (WHO) recommendations and other guides and regulations, and with comfort limits or health risks for the user of the space [57].

In this context, according to Dimitroulopoulou et al. [57], who compiled WHO and national guidelines to establish relevant indoor comfort levels for the temperature parameter, they distinguished these values into the following three ranges: (i) with an intermediate recommended temperature range of 20 to 26 °C, indicating ideal comfort; (ii) with a range of 16 to 20 °C, which is still within a comfortable range in the winter months; and (iii) with a range of 26 to 28 °C for the summer months. This results in a color representation on the label element associated with the object representing the sensor, varying between blue–yellow–green–yellow–red according to the temperature parameter. For relative humidity, the optimal % RH, for indoor air, considering health and work performance, is between 40% RH and 60% RH [58]. Also, according to the WHO’s published guidelines for dampness and mold [59], relative humidity (RH) above 80% supports significant growth of selected microorganisms; similarly, according to ASHRAE Standard 55 [60], relative humidity over 80% is acceptable if low temperatures occur. Consequently, within the established health safety parameters of 40 to 80% RH, the tag will display green; otherwise, it will be red. The WHO has not developed guidelines for indoor CO_2_ concentrations; however, health standards (such as HSE and ACGIH) set the CO_2_ concentration limits in workplaces at 5000 ppm for health and safety. Of note, the comfort-based value of 1000 ppm is described in the ASHRAE standard 62, but this was removed due to recurrent misinterpretations [61]. However, according to Lowther et al. [62], the range of 1000–1500 ppm represents acceptable or moderate indoor air quality (IAQ), and concentrations >1500 ppm represent poor IAQ. For the current work, extreme values of 5000 ppm (as a health and safety limit) and 1500 ppm (as an IAQ limit) are highlighted in the tag; therefore, if the value of the eCO_2_ (equivalent CO_2_) parameter is above this limit, then the tag will display red, if it is between 1500 and 5000 ppm, then the tag will display yellow, and if below this, the tag will display green. In Figure 9, the sensor position and associated tags can be viewed, allowing a special understanding of sensor data that is otherwise not possible with a schedule.

**Figure 10:**
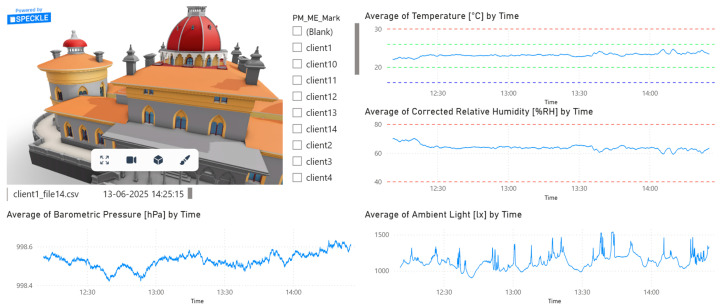
Power BI dashboard, for OMRON client 1 sensor environmental data, with Speckle Power BI viewer. Blue line is sensor data, red dash line represents heath safety threshold, and green dash line indicates comfort threshold.

Since this approach is limited by the level of information that can be interpreted from the sensor, a dashboard for each sensor was created to present more data (Figure 10). Also, using a URL parameter in the HBIM model, this dashboard would remain connected to the element. Likewise, if the dashboard were to be used independently, having a graphical representation of the model would be important. Therefore, the Speckle platform was explored for sharing models through a cloud-based approach due to its Revit connector and Power BI viewer. This method is advantageous for data consultation due to its interactive dynamic features, which allow the extraction of relevant data with ease; this is difficult to achieve with raw CSV data files.

The Power BI dashboard was built to integrate information from sensors in an intuitive manner, which is useful for building managers when accessing live strain on the conditions of buildings under management (e.g., too much humidity, CO_2_, or high temperatures) so that they can perform corrective measures, thus increasing the durability of the building. Additionally, pattern recognition can be used to implement proactive management or to stabilize conditions over time.

The data collected in this campaign provide a better understanding of the palace; however, a larger dataset is needed to identify problems and potential interventions. Also, due to the characteristics related to the current use of the palace, where it is operating as an open exhibition space without mechanical climatization, in addition to permanent users remaining in the space, and always having the doors open, the temperature and relative humidity values will continue to remain outside comfortable levels during peak periods of summer and winter.

## 5. Conclusions

This study presented and validated an integrated methodology for implementing a low-cost multivariable wireless sensor network (WSN) in a heritage building and linking it to an HBIM-based environment, contributing toward a Digital Shadow framework. The application to Monserrate Palace demonstrated both the feasibility and the practical challenges of deploying scalable, minimally invasive monitoring solutions in complex historic structures.

The experimental calibration highlighted the inherent limitations of affordable sensors, particularly in terms of accuracy, precision, and signal noise. Environmental monitoring revealed systematic bias relative to a reference instrument. Initial offsets reached up to approximately 2–3 °C for temperature and above 20% RH for relative humidity in some positions. However, after linear offset and gain correction, the RMSE was significantly reduced (e.g., to approximately 0.2 °C for temperature and 0.6% RH for relative humidity), demonstrating that, despite their lower intrinsic accuracy, these sensors can provide reliable trend monitoring when properly calibrated. For illuminance, higher dispersion and sensitivity to peak values were observed, reflecting both accuracy limits (±100 lx) and positioning effects. In vibration monitoring, the internal noise typical of MEMS accelerometers requires careful definition of trigger thresholds to avoid false activations, particularly on flexible wooden floors.

From an implementation perspective, key constraints were identified, including Wi-Fi attenuation caused by thick masonry walls, power supply instability, clock synchronization issues, data gaps during mode switching, and limitations in file size management. These operational challenges directly influenced data continuity and storage efficiency, reinforcing the importance of robust infrastructure planning in heritage environments.

The integration of monitoring data into the HBIM model, combined with dashboard visualization tools, enabled a centralized and structured data environment that enhances interpretation, supports condition assessment, and facilitates informed decision-making. By associating data with specific model elements, the methodology strengthens traceability, reduces redundancy, and improves operational efficiency in heritage management. The editable and updatable nature of BIM further supports long-term data accumulation and progressive knowledge enrichment.

Nevertheless, reliance on proprietary software and third-party cloud services raises concerns regarding interoperability, long-term accessibility, and technological obsolescence. While proprietary tools enable rapid implementation, future developments should prioritize more robust database infrastructures and open-source or interoperable solutions to ensure scalability and durability.

Overall, the results demonstrate that low-cost IoT-based monitoring, when supported by systematic calibration and structured digital integration, constitutes a viable and scalable strategy for heritage conservation and management. Future work should focus on long-term statistical analysis of accumulated datasets, improved power resilience of sensor nodes, migration to dedicated time-series databases, exploration of open-source solutions, and further advancement toward Digital Twin implementations.

## Figures and Tables

**Table 1:** OMRON 2JCIE-BU environment sensor (USB type) extractable parameters. In black values from sensors, and in grey values calculated from the measured quantities.

Sensor Type	Measurable Range	Accuracy	Unit
Temperature	−10 to 60 °C	±2 °C	0.01 °C
Relative humidity	30 to 85% RH	±5% RH	0.01% RH
Ambient light	10 to 2000 lx	±100 lx	1 lx
Barometric pressure	700 to 1100 hPa	±4 hPa	0.001 hPa
Sound noise	40 to 94 dB	-	0.01 dB
eTVOC (equivalent Total Volatile Organic Compound)	0 to 32,467 ppb	-	1 ppb
eCO_2_ (equivalent CO_2_)	400 to 32,467 ppm	-	1 ppm
Discomfort index ^1^	-	-	0.01
Heat stroke ^2^	-	-	0.01 ° C
Acceleration	−2000 to 2000 gal	-	0.1 gal
SI value ^3^	-	-	0.1 kine
PGA ^4^	-	-	0.1 gal
Seismic intensity ^5^	-	-	0.001

Performance values are obtained based on the various conditions of individual tests, and do not guarantee the values obtained under combined conditions for rated values and performance values. ^1^ Discomfort index: Expresses the heat and humidity of summer in a quantitative manner. It is calculated using temperature and humidity. ^2^ Heat stroke: Expresses the risk of heat stroke in a quantitative manner. It is calculated using temperature and humidity. ^3^ SI value: An index that expresses the effect that a certain vibration has on a structure. It has a correlation with seismic intensity. It is calculated from the acceleration values of two horizontal axes. ^4^ PGA: Peak acceleration value of a certain interval. It is calculated by combining the acceleration values of two horizontal axes. ^5^ Seismic intensity: A value correlated with seismic intensity that is calculated using the SI value.

**Table 2:** Characteristics of temperature, relative humidity, and ambient light sensors for ONSET HOBO U12-012 and OMRON 2JCIE-BU.

		HOBO U12-012	OMRON 2JCIE-BU
Sensor Performance	Resolution	12-bit	16-bit
	Range	~10.76 to 32,292 lx −20 to 70 °C 5 to 95% RH	10 to 2000 lx −10 to 60 °C 30 to 85% RH
	Accuracy	- ±0.35 °C from 0° to 50 °C ±2.5 from 10 to 90% RH	±100 lx ±2 °C ±5% RH
	Resolution	- 0.03 °C at 25 °C 0.05% RH	1 lx 0.01 °C 0.01% RH
	Voltage	0 to 2.5 V (VDC)	5 V (USB power supply)
	Data storage	Local Memory	Local Memory and IoT
Environmental Restrictions	Temperature and humidity range for best operation	−20 to 70 °C 0 to 95% RH (non-condensing)	−10 to 60 °C 30 to 85% RH 0 to 50 °C (Raspberry Pi)
	Size of sensor	58 mm × 74 mm × 22 mm 46 g	29 mm × 15 mm × 7 mm (sensor) 75 mm × 65 mm × 22 mm (box) 2.9 g (sensor) + 29 g (Raspberry Pi)
Economic Issues	Sensor cost	≈253 €	≈145 € 106 € (sensor) + 24 € (Raspberry Pi) + 4 € (SD) + 6 € (power cable) + 2.60 € (box)
	Market availability	N	Y (End of Life)
	Easy installation	Y	Y
	Need for accessories such as data acquisition systems, electric current, wiring, etc.	N	Y (no integrated battery)

## Data Availability

The original contributions presented in this study are included in the article. Further inquiries can be directed to the corresponding author.
